# Postnatal Development and Distribution of Sympathetic Innervation in Mouse Skeletal Muscle

**DOI:** 10.3390/ijms19071935

**Published:** 2018-07-01

**Authors:** Tatjana Straka, Veronica Vita, Kaltrina Prokshi, Sarah Janice Hörner, Muzamil Majid Khan, Marco Pirazzini, Marion Patrick Ivey Williams, Mathias Hafner, Tania Zaglia, Rüdiger Rudolf

**Affiliations:** 1 Institute of Molecular and Cell Biology, Mannheim University of Applied Sciences, 68163 Mannheim, Germany; t.straka@hs-mannheim.de (T.S.); rina90pro@web.de (K.P.); hoerner@kooperationen.hs-mannheim.de (S.J.H.); muzamil.m.khan@embl.de (M.M.K.); marion@g.clemson.edu (M.P.I.W.); m.hafner@hs-mannheim.de (M.H.); 2 Interdisciplinary Center for Neurosciences, Heidelberg University, 69120 Heidelberg, Germany; 3 Institute of Toxicology and Genetics, Karlsruhe Institute of Technology, 76344 Eggenstein-Leopoldshafen, Germany; 4 Venetian Institute of Molecular Medicine, 35129 Padua, Italy; verovita92@gmail.com (V.V.); marcopiraz@gmail.com (M.P.); tania.zaglia@unipd.it (T.Z.); 5 Department of Biomedical Sciences, University of Padua, 35131 Padua, Italy; 6 Department of Cardiac Thoracic and Vascular Sciences, University of Padua, 35128 Padua, Italy

**Keywords:** endplate, neuromuscular junction, sympathetic neuron, tyrosine hydroxylase, neuropeptide Y

## Abstract

Vertebrate neuromuscular junctions (NMJs) have been conceived as tripartite synapses composed of motor neuron, Schwann cell, and muscle fiber. Recent work has shown the presence of sympathetic neurons in the immediate vicinity of NMJs and experimental and clinical findings suggest that this plays an eminent role in adult NMJ biology. The present study examined the postnatal development and distribution of sympathetic innervation in different muscles using immunofluorescence, confocal microscopy, and Western blot. This demonstrates the proximity of sympathetic neurons in diaphragm, extensor digitorum longus, tibialis anterior, soleus, and levator auris longus muscles. In extensor digitorum longus muscle, sympathetic innervation of NMJs was quantified from perinatal to adult stage and found to increase up to two months of age. In diaphragm muscle, an extensive network of sympathetic neurons was prominent along the characteristic central synapse band. In summary, these data demonstrate that an elaborate sympathetic innervation is present in several mouse skeletal muscles and that this is often next to NMJs. Although the presence of sympathetic neurons at the perisynaptic region of NMJs increased during postnatal development, many synapses were already close to sympathetic neurons at birth. Potential implications of these findings for treatment of neuromuscular diseases are discussed.

## 1. Introduction

Congenital myasthenic syndromes (CMS) are neuromuscular transmission disorders that are due to mutations in one of several components needed for the function or maintenance of the synaptic apparatus of neuromuscular junctions (NMJs), such as choline acetyl transferase, agrin, or docking protein 7 (DOK-7) [1]. These mutations result in fatigable muscle weakness which can be partially treated by different drugs, depending on the gene affected and the type of mutation. In recent years, sympathicomimetic drugs, such as ephedrine or salbutamol, have proven to be rather effective in many CMS patients, but the underlying mechanisms of action have remained unclear [2]. A potential link of these clinical findings to NMJ biology has been the recent observation of extensive interaction between sympathetic neurons and NMJs [3,4]. Indeed, in adult mouse extensor digitorum longus and diaphragm muscles, sympathetic neurons were found to form ample networks between motor neurons, blood vessels, and muscle fibers [4]. In particular, most NMJs were in immediate proximity to tyrosine hydroxylase (TH)-positive sympathetic neurons in soleus and extensor digitorum longus muscles. Moreover, functional in vivo-imaging using fluorescent biosensors expressed in tibialis anterior muscles showed rapid activation of postsynaptic beta-adrenergic receptor and 3′,5′-cyclic adenosine monophosphate (cAMP) signaling as well as nuclear import of the transcriptional co-activator, peroxisome proliferator-activated receptor gamma coactivator 1-alpha, upon electric stimulation of the ganglia mediating sympathetic outflow to the hindlimbs [4]. Local chemical sympathectomy led to massive muscle atrophy and a decay of NMJ integrity and function. These features were similar to those observed in a CMS mouse model and were rescued by simultaneous treatment with a sympathicomimetic drug [4]. With this in mind, one might ask, if sympathetic innervation of skeletal muscle and NMJs was not addressed before. Indeed, the first accounts on a potential ‘dual innervation’ by myelinated and non-myelinated fibers of NMJs were published already in the early twentieth century [5,6,7], but for different reasons, including lack of molecular specificity and functional proofs, these studies were later dismissed [8] and largely forgotten. Sporadic studies reported on the distribution of sympathetic neurons in skeletal muscle as a whole [9] or at muscle spindles [10], on the dual innervation of special NMJs in the esophagus [11,12], or on the presence of the catecholaminergic neuron marker, TH, opposite to acetylcholine receptor (AChR) stainings in mouse and human muscle cross-sections [13,14]. The latter studies, though, interpreted their findings such that the cholinergic motor neuron itself might have TH activity rather than suggesting the presence of another, sympathetic, neuron at the perisynaptic region. Such a conclusion was reasonable in the framework of the long-held concept of a tripartite structure of NMJs and given that the observations were made on muscle cross sections, which do not allow the visualization of axons approaching the synapse. This was different in the most recent study, which used optical tissue clearing in combination with reporter mice expressing a fluorescent protein under dopamine beta-hydroxylase promoter control and which showed axons different to those of lower motor neurons approaching the NMJs [4]. Additional recent work argues in favor of an important role of sympathetic innervation for muscle trophic status [15] and in the context of CMS [1,16,17] and other neuromuscular diseases including spinal muscular atrophies and amyotrophic lateral sclerosis [18]. Thus, the sympathetic innervation of muscle, and of NMJs in particular, is a new field of research and nothing is known so far about its developmental aspects. In the light of early-onset neuromuscular disorders, such as certain types of CMS, the early postnatal development of the sympathetic-NMJ relationship is especially relevant. This was addressed in the present work and, in addition, the list of muscles showing sympathetic innervation at NMJs was further increased.

## 2. Results

### 2.1. Sympathetic Innervation is Widely Distributed in Hindleg Muscles

**Figure 1:**
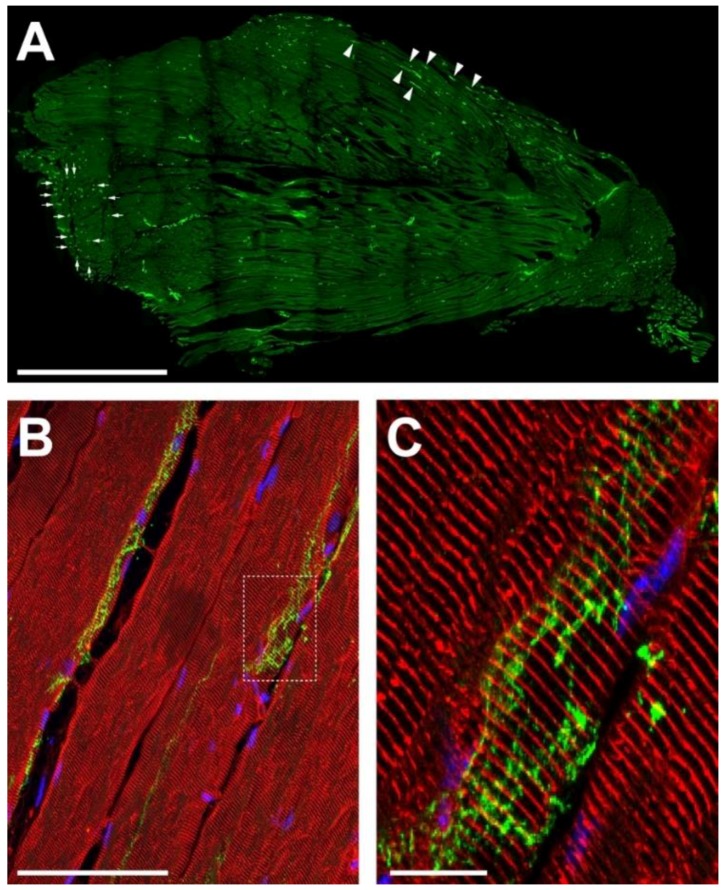
Sympathetic innervation is richly developed in mouse hindleg muscle. Tibialis anterior muscles of adult wildtype mice were cryosectioned longitudinally, then stained with antibodies against TH (**A**–**C**, green) and, in addition, against α-actinin (**B**,**C**, red). Nuclei were stained with 4′,6-diamidino-2-phenylindole (DAPI; **B**,**C**, blue). Pictures show single confocal sections, (**C**) depicts the detail in the boxed region of (**B**). Arrowheads and arrows in (**A**) indicate alignment of sympathetic neurons with longitudinally and vertically cut muscle fibers, respectively. Scalebars show 500 µm, 50 µm, and 5 µm in (**A**–**C**), respectively.

First, we studied the general distribution of sympathetic innervation in the tibialis anterior hindleg muscle. Longitudinal sections were prepared and stained with an antibody specific for the sympathetic neuron marker, TH. As shown in Figure 1A, TH immunofluorescence signals were visible along the entire extension of the muscle belly. Partially, longer stretches of TH-positive signals were found to run along individual muscle fibers if those were nicely cut in length as indicated for some examples by arrowheads in Figure 1A. In other regions, where muscle fibers were rather seen in cross-section, TH-positive signals appeared as dots (arrows in Figure 1A). This suggests, that TH-positive axons generally align for some distance along the muscle fibers. This was confirmed by further analyses, where slices were additionally stained with an antibody against α-actinin to highlight the muscle fiber sarcomeres (Figure 1B,C). TH-positive signals were seen to run in anastomosing patterns along the outside of the fibers. In general, the data show that sympathetic innervation is richly developed in mouse tibialis anterior muscle and that the sympathetic neurons might communicate at many places with muscle fibers.

### 2.2. Sympathetic Innervation Contacts NMJs in Different Muscles

**Figure 2:**
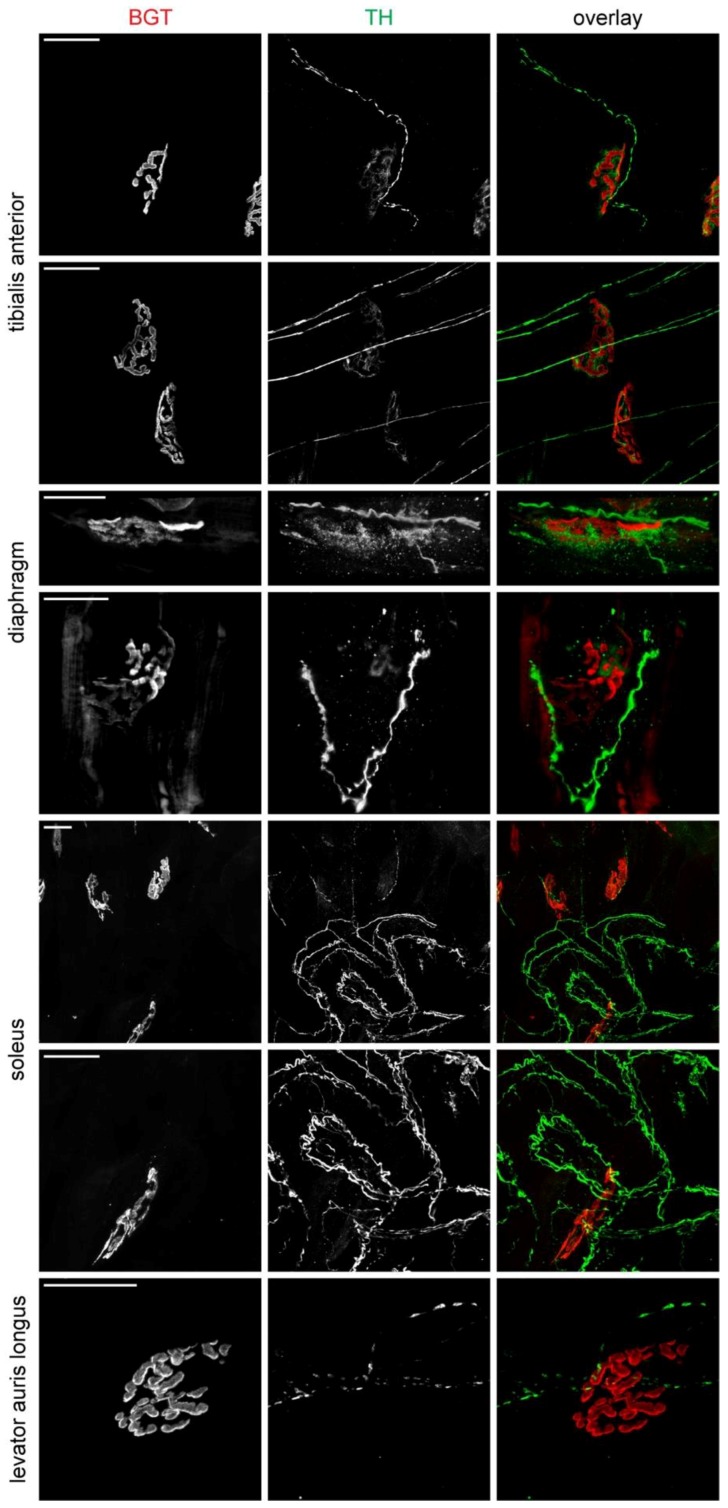
Sympathetic innervation approaches NMJs in various skeletal muscles. Diaphragm muscle was from P30, tibialis anterior, soleus, and levator auris longus muscles were from P90 wildtype mice. Except for diaphragm muscle, which was prepared as a whole mount, fiber bundles were teased from these muscles as indicated and stained for NMJs (BGT, red in overlays) and sympathetic neurons (TH, green in overlays). Images show maximum z-projections of confocal image stacks. Scalebars, 20 µm.

Next, we concentrated on the interaction between sympathetic innervation and NMJs in different adult muscles, i.e., tibialis anterior, diaphragm, soleus, and levator auris longus. Except for diaphragm, fiber bundles were teased and co-stained with an anti-TH antibody and fluorescent α-bungarotoxin (BGT) for labelling sympathetic neurons and NMJ postsynapses, respectively. Confocal microscopy of these samples confirmed a rich innervation in all assessed muscles and showed that most NMJs were in very close proximity to at least one ramification of a sympathetic neuron (Figure 2). The formation of plaque-like TH-positive structures directly underlying the postsynaptic apparatus, as it was described previously for extensor digitorum longus muscle [3,4], was also evident in tibialis anterior muscle (Figure 2, upper two panel rows) and diaphragm (Figure 2, third panel row), less clear in soleus (Figure 2, middle two panel rows) and apparently absent in levator auris longus (Figure 2, lower panel row). This suggests, that although the immediate vicinity of NMJs to sympathetic ramifications appears to be a general feature, the precise form of morphological interaction may vary between different muscle types. Interestingly, the TH-signals in the plaque-like structures underneath the NMJs, as observed in the tibialis anterior (Figure 2, upper two panel rows) and diaphragm (Figure 2, panel rows 3 and 4), was rather outlining the postsynaptic BGT staining. This is in contrast to lower motor neuron signals that perfectly match the postsynapse (for review see e.g., [19]).

### 2.3. Sympathetic Innervation of NMJs Increases during Postnatal Development

**Figure 3:**
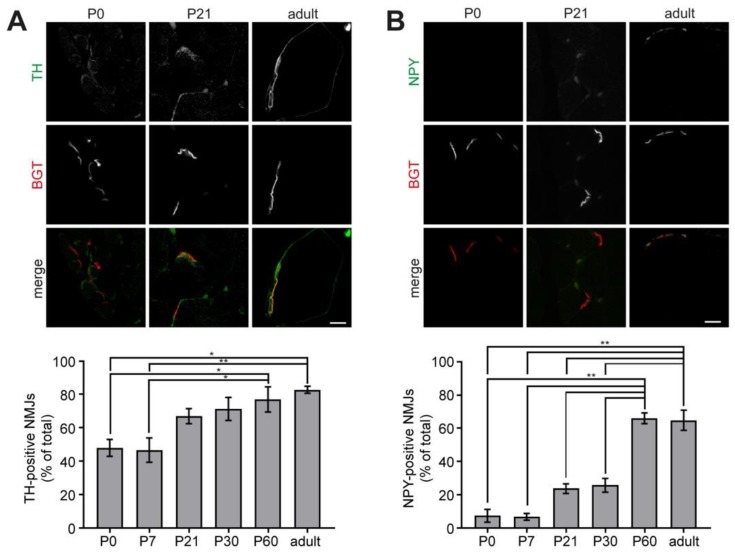

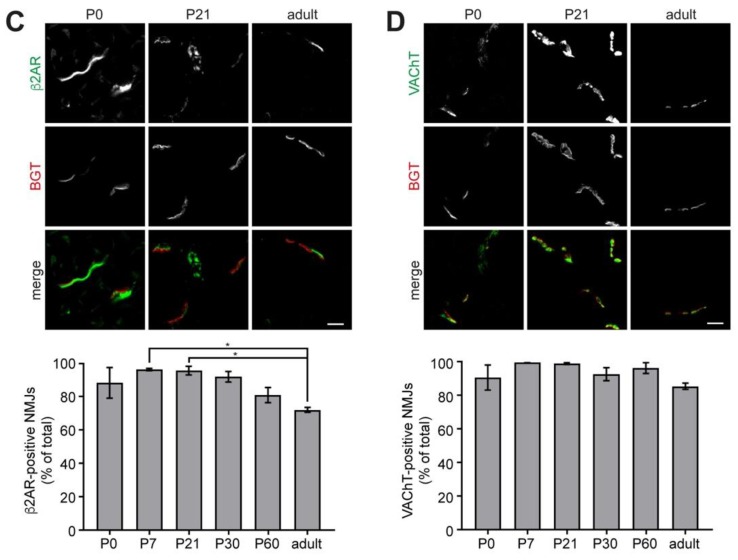
Sympathetic innervation of NMJs increases during postnatal development in mouse hindlimb muscle. Mouse extensor digitorum longus muscles from animals aged zero days (P0) to six months (adult) were sampled, fixed, transversally sectioned, and stained for NMJs (BGT, red in merges) and various marker proteins (green in merges). Therefore, antibodies against TH (**A**), NPY (**B**), β2-adrenergic receptor (**C**, β2AR), and vesicular acetylcholine transporter (**D**, VAChT) were employed. Labeled sections were imaged by confocal microscopy and colocalization between BGT-signals and immunolabelling was determined. Fluorescence micrographs show single optical sections of representative tissue slices from P0, P21, and adult. Scalebars, 10 µm. Bar graphs show quantitative analysis of immunopositive NMJs as percent of all analyzed NMJs (mean ± SEM; *n* = 3). In total, more than 8400 NMJs were analyzed. * *p* < 0.05, ** *p* < 0.01.

To address the postnatal development of the interaction between sympathetic neurons and NMJs, we performed immunofluorescence and Western blot analyses. For the immunofluorescence study, extensor digitorum longus muscles were harvested from wildtype mice at different ages ranging from P0 to adult, cross-sectioned, and stained with BGT for NMJs in addition to either anti-TH or anti-neuropeptide Y (NPY) antibodies to mark sympathetic neurons; with anti-β2-adrenergic receptor antibodies to label target structures of sympathetic neurons, or anti-VAChT antibodies against cholinergic motor neurons as a positive control. Samples were then imaged using confocal microscopy and quantitatively analyzed for colocalization using ImageJ. Figure 3 shows representative confocal slices for each of these stainings at P0, P21, and adult. Quantitative analysis revealed that the amount of NMJs positive for the sympathetic neuron markers significantly increased from about 40% in the case of TH and less than 10% for NPY at P0 to almost 90% (TH) and 60% (NPY) in adult muscles (Figure 3A,B). Conversely, the number of NMJs positive for β2-adrenergic receptor immunostaining remained constantly high at around 80 to 90% up to P30 and slightly decreased to about 70% in the adult (Figure 3C). As expected, the percentage of VAChT-positive NMJs did not vary significantly and ranged at all analyzed stages between 90% and 100% (Figure 3D).

**Figure 4:**
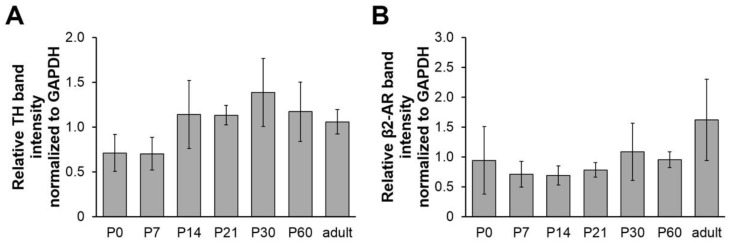
Total amounts of TH and β2AR in hindleg muscles do not vary significantly during postnatal development. Mouse tibialis anterior muscles from animals aged zero days (P0) to six months (adult) were sampled, lysed, and subjected to SDS-PAGE followed by Western blot analysis. In each lane, the same amount of protein was loaded. Western blot used antibodies against TH (**A**) or β2AR (**B**). Glyceraldehyde 3-phosphate dehydrogenase (GAPDH) was employed as a loading control. Bar graphs depict TH or β2AR band intensities relative to their corresponding GAPDH band (mean ± SEM; *n* = 4 mice for each time point).

Furthermore, using Western blot analysis of lysates from tibialis anterior muscles, we also studied the postnatal whole-muscle expression profile of TH and β2-adrenergic receptors (β2AR). In contrast to the immunofluorescence data, no significant variation in the amount of TH or β2AR was detected throughout the different samples (Figure 4). In summary, the combination of immunofluorescence and Western blot data suggests, that although the overall sympathetic innervation density seemed to remain unaltered during the postnatal development in the studied hindleg muscles, NMJs became increasingly approached by TH-positive sympathetic neurons. Conversely, in the same samples, β2AR total amounts and distribution at NMJs were similar during the entire observation period.

### 2.4. Sympathetic Innervation in Mouse Diaphragm Aligns with the Synapse Band

**Figure 5:**
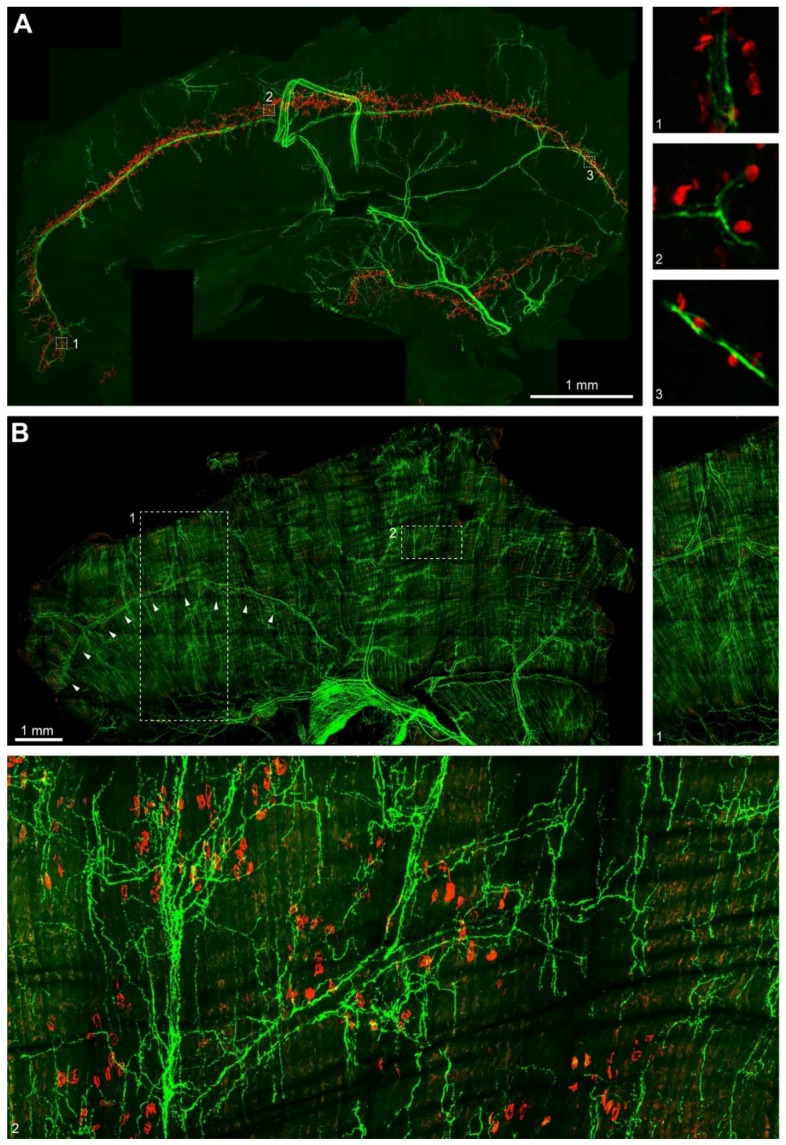
Sympathetic innervation of mouse diaphragm muscle gains complexity during postnatal development. Mouse diaphragm muscles from P0 (**A**) and P30 (**B**) mice were fixed, stained with BGT against NMJs (red) and antibodies against TH (green). Then, muscles were imaged by high-resolution tile scanning confocal microscopy with a lateral resolution of 758 nm and a vertical step size ranging between 1 and 1.2 µm (P0) and 7 µm (P30). Image acquisition was followed by deconvolution. Large pictures in (**A**,**B**) depict maximum z-projections of hemidiaphragms. Insets 1, 2, 3 show single optical sections (**A**) or enhanced details (**B**) from correspondingly numbered boxed regions in the overview images. Arrowheads in (**B**) indicate a zone, where a main branch of the phrenic artery is lined by TH label.

Finally, we investigated the distribution of sympathetic innervation in diaphragm muscle. To that end, diaphragms from newborn and one-month old mice were taken, fixed, and stained with BGT and antibodies against TH. After embedding, whole mounts were imaged with confocal microscopy using a Leica HC PL APO 20x/0.75 IMM CORR objective with voxel sizes of 758 nm in xy and 1–1.2 µm (P0) or 7 µm (P30) in z. Tile stacks were then stitched and deconvoluted. Figure 5A shows a z-projection of a P0 hemidiaphragm with the well-known distribution of NMJs (red spots) in two discrete synapse bands in the costal and crural muscle domains. Notably, although TH-positive axons were found throughout the hemidiaphragm, the highest density of these nerve fibers was present along the synapse bands. The strongest TH-signals were those along the phrenic arteries, but finer processes ran into proximity of most NMJs. Moreover, also intercostal arteries were nicely outlined by TH fluorescence signals. To get a better idea of the local relationship between the localization of sympathetic axons and NMJs, single confocal sections of blow-ups from boxed regions 1, 2, and 3 in the overview are depicted in the small insets numbered accordingly in Figure 5A. These show that most NMJs were found in the immediate vicinity of TH-fluorescence positive ramifications. Inspection of P30 diaphragms revealed an increasing complexity of sympathetic innervation (Figure 5B). Although many NMJs were still found to align on bands close to the phrenic arteries and their accompanying sympathetic neurons (in particular on the ventral half of the diaphragm, see arrowheads in Figure 5B), the widening NMJ bands in other parts of the muscle were approached by numerous fine anastomoses of sympathetic projections. These ramifications mostly originated from the blood-vessel aligned TH-positive processes. As depicted in insert 1 to Figure 5B, sympathetic projections from intercostal and phrenic blood vessels were crossing at the center of the costal diaphragm domain and then extended in a dense meshwork of thin axons running along muscle fibers for hundreds of microns. Conversely, the sympathetic innervation was much sparser in the tendon region (see lower part of insert 1 in Figure 5B). Insert 2 of Figure 5B depicts a central part of the costal domain and demonstrates the high density of sympathetic innervation at P30. The alignment of TH-positive axons with blood vessels is here as evident as the emanation of finer branches from the principal projections. It can be noted, that the matrix of sympathetic projections enables that nearly all NMJs are at close distance to one or more of these.

**Figure 6:**
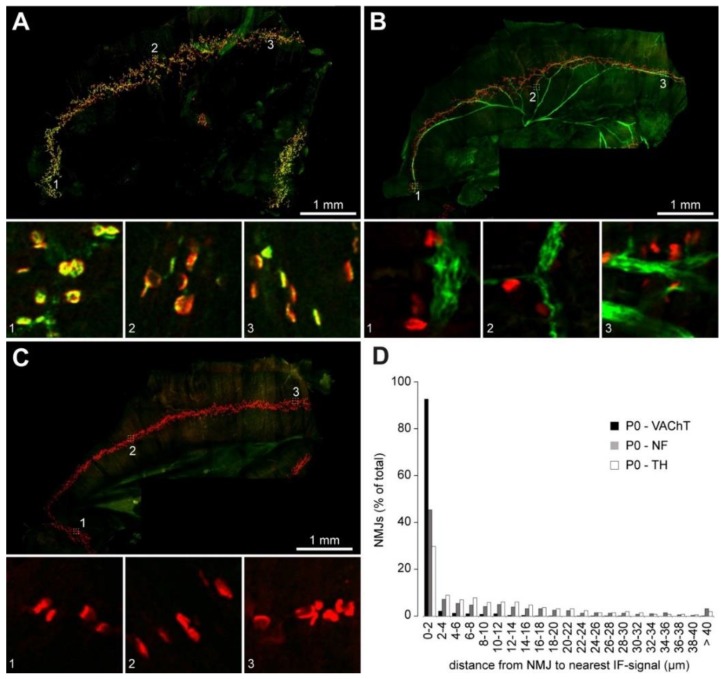
Quantitative analysis confirms interaction of NMJs with sympathetic ramifications in postnatal diaphragm muscles. Mouse diaphragm muscles from P0 mice were fixed, stained with BGT against NMJs (red signals in **A**–**C**) and antibodies (green signals in **A**–**C**) against VAChT (**A**) or neurofilament L (**B**). In (**C**), primary antibody was omitted. Then, muscles were imaged by high-resolution tile scanning confocal microscopy with a lateral resolution of 758 nm and a vertical step size ranging between 1 and 1.2 µm. Image acquisition was followed by deconvolution. Large pictures in (**A**–**C**) depict maximum z-projections of hemidiaphragms. Insets 1–3 show single optical sections from the correspondingly numbered boxed regions in the overview images. (**D**) shows a distribution plot indicating the distance of individual NMJs to the nearest corresponding immunofluorescence signal. Image data for P0—TH were as shown in Figure 5A. In total, 5986 NMJs were analyzed.

To gain more concrete insights into this feature, we carried out a quantitative assessment of how close NMJs really were to the sympathetic neurites. As a positive control, we first used a proximity calculation between VAChT immunostaining and NMJs. Since, in skeletal muscle, VAChT is almost exclusively present in all cholinergic presynapses, this should indicate the quality of our data processing. Thus, P0 diaphragms were stained, imaged (Figure 6A) and then analyzed using the DiAna plugin for ImageJ [20]. As illustrated in the distribution plot in Figure 6D, more than 90% of NMJ postsynapses as identified by BGT were found at less than 2 µm to the next VAChT immunofluorescence signal. This shows that the segmentation and analysis tool were sufficient to address the proximity analysis, at least for plaque-like fluorescence signals such as those obtained with VAChT and BGT staining. However, the TH staining tended to become less intense in the finer ramifications and plaque-like TH-positive structures as found in tibialis anterior muscles and P30 diaphragms (Figure 2) were not seen in the P0 diaphragms. Accordingly, only about 30% of NMJs were found in a range of less than 2 µm diameter to the next TH signal (Figure 6D) in P0 diaphragms. Therefore, we decided to compare these values to another positive control, i.e., neurofilament L, a filamentous axonal marker, which appeared to primarily stain motoneurons in our preparations (Figure 6B). The quantitative proximity data retrieved with this marker were much more like those of TH (Figure 6D). In conclusion, these data indicate that many NMJs are in close vicinity to TH-positive neurons in the diaphragm.

## 3. Discussion

Skeletal muscle, which is composed of many interweaved tissue components such as skeletal muscle fibers, blood vessels, and nerve and glial cells, is clearly a target for sympathetic activity, be it in the context of vasomotor control [21,22] or the regulation of muscle force [23,24,25]. However, the dual possibility of regulation by either the hormonal or the neural branch of the sympathetic nervous system has led to a profound ambiguity on the precise mechanisms and origins of sympathetic stimuli in many cases. In particular, sympathetic innervation of skeletal muscle fibers has been widely neglected and, in general, the presence and distribution of sympathetic neurons in skeletal muscles has been rarely studied [9,26]. To our knowledge, an overview on the sympathetic innervation in a whole mount muscle has been completely missing so far. The lack of knowledge is even more eminent with respect to the interaction of sympathetic neurons and NMJs. Indeed, NMJs are still the classical example for a tripartite synapse [27] composed of and regulated by three cell types: cholinergic lower motor neuron, skeletal muscle fiber, and terminal Schwann cell. Although it remains undoubted, that these are the major components of NMJs, recent work has added sympathetic neurons as a potential fourth candidate that is frequently found in the immediate vicinity of NMJs in certain muscles, that appears to be functionally linked with the NMJ postsynaptic part, and to be crucial for the maintenance of the entire synaptic apparatus [4]. This latter study triggered a plethora of questions, which need to be examined in future investigations with two of these, i.e., which muscles exhibit an NMJ—sympathetic neuron connection and how is its postnatal development, being addressed here.

In combination with earlier work [3,4], the current investigations could increase the list of muscles with an apparent strong relationship between NMJs and sympathetic neurons to the following: diaphragm, extensor digitorum longus, gastrocnemius, levator auris longus, soleus, and tibialis anterior. Each of these muscles displayed an ample distribution of sympathetic neurites over large ranges of the muscle and many NMJs were near such a ramification. However, only diaphragm, extensor digitorum longus, gastrocnemius, and tibialis anterior muscles displayed a clear TH-positive extension of the sympathetic neurite underlying the NMJ postsynapse in a plaque-like manner. It needs to be seen in further investigations if this feature has any relevance for functional interaction between neuron and muscle, and why some muscles exhibit this kind of connection and others do not. Given that both, slow (like diaphragm) and fast muscles (like tibialis anterior) showed plaques, it seems unlikely, that it is linked to muscle fiber type. This was also corroborated by a preliminary fiber typing study, which showed that sympathetic processes were present along type I, IIa, and IIb myosin-positive muscle fibers (Figure S1). Furthermore, given that these TH-positive signals were regularly outlining the postsynaptic BGT-signals, one would expect to consistently find profiles that are typical for sympathetic neurons with sparse small synaptic vesicles and some large dense core vesicles at the rims of synaptic NMJ boutons. In addition, it would be interesting to study the precise morphological position of sympathetic neurons in relation to postsynaptic junctional folds, motor neurons, and Schwann cells by means of super-resolution microscopy or correlative light-electron microscopy.

The analysis of the postnatal development of the sympathetic neuron—NMJ interaction showed a gradual increase of innervation density from P0 to adult for two sympathetic neuron markers, i.e., TH and NPY. However, while enrichment of TH-signals juxtaposed to the BGT staining was already present in many NMJs at birth, NPY immunoreactivity appeared much later at around week three at that place. It would be important to obtain insights concerning the relevance of this finding. For example, it would be interesting to know, if there are potentially different functions of catecholamines versus NPY at the level of the NMJ and why NPY is appearing later in postnatal development than TH. Conversely, β2AR staining was strong at P0 and slightly decreased at later ages. Clearly, β2AR immunofluorescence signals were not only present at the NMJ site but were also found in other places. As previously observed [3], motor axons and blood vessels were often immunopositive and it will be interesting to study putative functions of a local sympathetic—β-adrenergic interplay.

To our knowledge, this study has delivered the first pictures of the distribution of sympathetic innervation across muscle whole mounts. In particular, the spreading of TH-signals in the diaphragm was very interesting. This showed an apparent intimate relationship of sympathetic axons with at least two major components of muscle tissue, i.e., blood vessels and NMJs. Indeed, although we here did not include direct blood vessel staining, it can be inferred from the general function of sympathetic neurons in regulating vasomotor activity [21,22,28] and earlier work on the distribution of arteries, veins, and capillaries in diaphragms [29,30] that the major TH-positive signals in our hemidiaphragm samples were lining up with the phrenic and intercostal arteries and their ramifications. Interestingly, compared to P0, the sympathetic innervation was more elaborate at P30. While at P0 most of the TH-positive fibers were confined to the large phrenic and intercostal blood vessels and did extend from there only roughly up to the border of the synapse band, TH-positive processes were also running along the entire sarcomeric region in P30 diaphragm. The co-alignment of the TH-positive signals with the NMJ synapse bands was intriguing and triggers open questions. These include, whether there is a special relationship between NMJs and macro- and microcirculation and if so, whether this finding is limited to diaphragm muscles. A preliminary analysis on cross sections of different hindleg muscles confirmed that TH-positive structures mostly contact both, NMJs and blood vessels (Figure S2), but did not yield a special vicinity correlation between NMJs and blood vessels. However, this certainly needs further consolidation.

Finally, the present study reveals an ample presence of sympathetic innervation and interaction with NMJs already at birth. Some neuromuscular disorders, including several forms of congenital myasthenic syndromes, set in perinatally and an increasing amount of clinical evidence show the effectiveness of sympathicomimetic drugs, such as ephedrine, salbutamol, or albuterol, for clinical treatment of certain types of these diseases [1,2,31]. Experimental data in rodent models corroborate these insights and point to an important function of sympathetic innervation for the maintenance of NMJs [4]. The present findings of an elaborate sympathetic plexus ready at birth but with augmenting complexity and higher interaction rate with NMJs are compatible with a role of this innervation for NMJ development as well and could indicate that early postnatal treatment with sympathicomimetics might be envisaged in certain cases of perinatal neuromuscular disorders.

## 4. Materials and Methods

### 4.1. Animals and Sample Preparation

In the current study, P0, P7, P14, P21, P30, P60, and adult C57BL/10J and C57BL/6J (Charles River) mice were used. Animals were maintained in a local animal facility and their use and care were approved by German and Italian authorities (Regierungspräsidium Karlsruhe, G-285/14, 11 June 2016 and local ethical committee and Ministry of Health–Ufficio VI, C54).

### 4.2. Western Blot

For Western blot analysis, *tibialis anterior* muscles were dissected, snap-frozen, lysed using lysis buffer (50 mM Tris-HCl, pH 8.0, 150 mM NaCl, 1% NP-40 [A1694; AppliChem, Darmstadt, Germany], 10% glycerol, 1 mM EDTA, 1 mM EGTA, 1× Roche^®^ phosphatase inhibitor cocktail [#88667; Roche, Mannheim, Germany], 1× Roche^®^ protease inhibitor cocktail [#88665; Roche, , Mannheim, Germany] and 0.5 mM PMSF [A0999; AppliChem, Darmstadt, Germany]), and subjected to SDS-PAGE followed by Western blot analysis. In each lane, equal amounts of protein were loaded. Chemiluminescence signals were obtained using an ECL system in combination with a Syngene G:Box Chemi XX6 chemiluminescence imager (Fisher Scientific, Schwerte, Germany). Western blot analysis employed the following antibodies: Rabbit anti-TH (Millipore AB152; 1:1000; Merck, Darmstadt, Germany), rabbit anti-β2AR (AB182136; 1:1000; Abcam, Cambridge, UK), mouse anti-GAPDH (MA5-15738; 1:10,000; Thermo Fisher Scientific, Darmstadt, Germany), goat anti-rabbit IgG (H + L) HRP (Jackson ImmunoResearch 111035003; 1:10,000; Dianova, Hamburg, Germany), and goat anti-mouse IgG (H + L) HRP (#32430; 1:10,000; Thermo Fisher Scientific, Darmstadt, Germany).

### 4.3. Immunofluorescence

Immunofluorescence analyses used the following antibodies: Rabbit anti-TH (Millipore AB152; 1:200 on sections; 1:100 on fiber bundles; 1:50 on whole mounts; Merck, Darmstadt, Germany), rabbit anti VAChT (#139103; 1:500 on sections; 1:200 on whole mounts; Synaptic Systems, Göttingen, Germany), rabbit anti-NPY (#11976; 1:200 on sections; Cell Signaling Technologies, Frankfurt am Main, Germany), rabbit anti-β2AR (sc-569; 1:200 on sections; Santa Cruz Biotechnology, Heidelberg, Germany), rabbit anti-NFL (#171002; 1:200 on whole mounts; Synaptic Systems, Göttingen, Germany), mouse anti-myosin heavy chain-type I (1:100, [32]); anti-myosin heavy chain-type IIa (1:100; [32]) and anti-myosin heavy chain-type IIb (1:100, [32]), goat anti-mouse AlexaFluor555 (Dianova, Hamburg, Germany), donkey anti-rabbit AlexaFluor 488 (1:1000 on sections; 1:200 on whole mounts (P0), 1:100 (P30); Thermo Fisher Scientific, Darmstadt, Germany). AChR were labeled with BGT-AlexaFluor 647 (Life Technologies B35450; 1:1,000 on sections; 1:200 on whole mounts (P0), 1:100 (P30); Thermo Fisher Scientific, Darmstadt, Germany), blood vessels were stained with FITC-conjugated lectin-B4 (VEC-FL-1201, [33]; 1:100; Biozol, Eching, Germany). (a) For fiber bundle analysis, muscles were harvested, fixed in 4% PFA for 5 min and small fiber bundles were dissected in sterile PBS under a stereomicroscope. Fibers were incubated in 50 mM NH_4_Cl for 30 min, followed by washing in PBS. Fibers were permeabilized in PBS supplemented with 0.5% Triton-X100 for 2 h at room temperature and incubated overnight with the primary antibody diluted in PBS supplemented with 2% BSA and 0.5% Triton-X100 at 4 °C. After three washes in PBS, fibers were incubated with the secondary antibody for 2 h at room temperature. At the end of the procedure, fibers were incubated with BGT for 2 h at room temperature at slow agitation. Fibers were then analyzed at the confocal microscope; (b) For immunofluorescence of cryosections, muscles were sampled, chemically fixed in 4% PFA (overnight, 4 °C), and cut in 8 µm thick slices. Sections were washed with 1× PBS (10 min), permeabilized with 0.1% Triton-X100/PBS (10 min), washed with 1× PBS (3 × 5 min), and blocked with 2% BSA/PBS (2 h, 4 °C). Then, sections were incubated with primary antibodies in 2% BSA/PBS (overnight, 4 °C). After washing with PBS (3 × 10 min), the slides were incubated with secondary antibodies and BGT in 2% BSA/PBS (1 h) followed by washing with PBS (3 × 10 min). Slides were embedded in Mowiol; (c) For immunofluorescence of whole mounts, diaphragms were chemically fixed in 4% PFA (24 h, 4 °C). For immunostaining a modified iDISCO tissue clearing and staining protocol was applied [34]. In brief, hemidiaphragms were dissected and incubated in blocking and permeabilization solution (BnP) composed of 1× PBS/1× PTwH (0.2% Tween in 1× PBS with 10 µg/mL heparin)/0.5% Triton X-100/10% (*vol*/*vol*) DMSO/6% (*vol*/*vol*) BSA (1× BnP) for 3 days followed by a 24 h-incubation in quenching solution (1× PBS/0.5% Triton X-100/20% DMSO/0.3 M glycine). The primary antibody was diluted in 1× BnP and incubated at 37 °C on an orbital shaker for 24 h (P0) or 4 days (P30). Then, hemidiaphragms were washed with 1× BnP for 4 days and incubated with the secondary antibody and BGT (diluted with 1× BnP) at 37 °C for 24 h on an orbital shaker. Before imaging, another 4 days of 1× BnP and 24 h of ddH_2_O washing was performed. Then, P0 hemidiaphragms were mounted on a glass slide and embedded using Mowiol. RI matching with glycerol was performed only in hemidiaphragms aged P30 and embedded using glycerol. If temperature was not specified, incubation was performed at room temperature.

### 4.4. Microscopy

Muscles were imaged with inverted Leica SP2 and SP8 (Leica Microsystems CMS, Mannheim, Germany) confocal microscopes equipped with HC PL APO 20× /0.75 IMM CORR, HC PL APO 63× /1.40 OIL CS2, and HC PL APO CS2 63× /1.2 W CORR objectives. Whole mount microscopy of hemidiaphragms used automated tile scan imaging with the Navigator module and automated stitching in the smooth mode with the Stitching module of Leica LAS-X 3.3.0 software suite. Projections of hemidiaphragms were rendered with the 3D Visualization module of Leica LAS-X 3.3.0 software suite.

### 4.5. Image Analysis

(a) Accumulation Analysis. The settings used at the Leica SP2 were 400 Hz scan frequency, 1024 × 1024-pixel resolution, two times line average, and a pinhole of 4 Airy Units. For further processing, the images were exported in TIFF format. Accumulation analysis was conducted using ImageJ freeware image processing software (https://imagej.nih.gov/ij/) as previously described [35]. Briefly, images of the BGT-AF647 channel were median filtered (1-pixel kernel) and thresholded from 30–255 grayscale values. The NMJ region in the BGT-AlexaFluor647 channel was outlined as a region of interest (ROI). In the AlexaFluor488 channel, the muscle fiber area corresponding to the previous selected NMJ region was defined. For both (NMJ region and muscle fiber), mean grey value and standard deviation were determined. The accumulation of the stained proteins within the NMJ region was counted as positive if the mean grey value within the NMJ region was higher than the mean gray value within the muscle fiber plus the standard deviation within the fiber. In formula: [(mean grey value NMJ) − (mean grey value fiber + standard deviation fiber)]; (b) Distance Analysis. For the analysis of the distance between the immunostaining signals (AlexaFluor488) and the NMJs (BGT-AlexaFluor647) hemidiaphragms were imaged with the following settings: 1024 × 1024 pixels, voxel size: 0.785 × 0.785 × 1 µm (TH), 0.785 × 0.785 × 1.04 µm (VAChT), 0.785 × 0.785 × 1.2 µm (neurofilament L and negative control), bi-directional scan speed 600 Hz, zoom 0.75, pinhole setting of 1 Airy Unit, and a frame average of 3. Deconvolution with Huygens Essential 15.10 software (https://svi.nl/HomePage) (RRID: SCR_014237) was performed using a maximum of 300 iterations of the Classic Maximum Likelihood Estimation algorithm with a theoretical PSF. Background correction employed automatic settings. The signal-to-noise ratio setting was set to 1 and the quality threshold to 0.05. For the distance analysis, the Fiji plugin “DiAna” was used [20]. In brief, “DiAna” is suited for 3D image segmentation and 3D distance analysis within 3D data sets. For the analysis of the edge-to-edge distance between BGT-AlexaFluor647 and the closest immunofluorescence (AlexaFluor488) signal, the following procedure was applied. First, images were intensity thresholded for binarization using the following intensity boundaries: BGT signals, 20–255; TH, 6–255; neurofilament L, 22–255; VAChT, 20–255. Then, the “classical segmentation” procedure was selected. For BGT signals, minimum and maximum object sizes of 50 and 20,000,000, respectively, were set. For all immunostainings, object sizes of 3–2,000,000,000 were set since the filamentous structure of TH and neurofilament L signals required a higher maximum object size. In the BGT channel, signals at x, y, and z edges were excluded.

## Supplementary Materials

Supplementary materials can be found at http://www.mdpi.com/1422-0067/19/7/1935/s1.

## Abbreviations

AChR	nicotinic acetylcholine receptor
BGT	α-bungarotoxin
BnP	blocking and permeabilization solution
β2AR	β2-adrenergic receptor
CMS	congenital myasthenic syndrome
GAPDH	glycerol aldehyde-3-phosphate-dehydrogenase
NMJ	neuromuscular junction
NPY	neuropeptide Y
PTwH	0.2% Tween in PBS with 10 µg/mL heparin
TH	tyrosine hydroxylase
VAChT	vesicular acetylcholine transporter
