# Supplementary material for: Postnatal Development and Distribution of Sympathetic Innervation in Mouse Skeletal Muscle
Source: Int J Mol Sci. 2018 Jul 1;19(7):1935. doi: 10.3390/ijms19071935 (PMC6073285; doi:10.3390/ijms19071935)
Supplement: Supplementary file 1 [file ijms-19-01935-s001.zip › ijms-318366-SI.pdf]

Supplementary Figures to manuscript ijms-318366_rev

Postnatal development and distribution of sympathetic innervation in mouse skeletal muscle

by Straka et al.

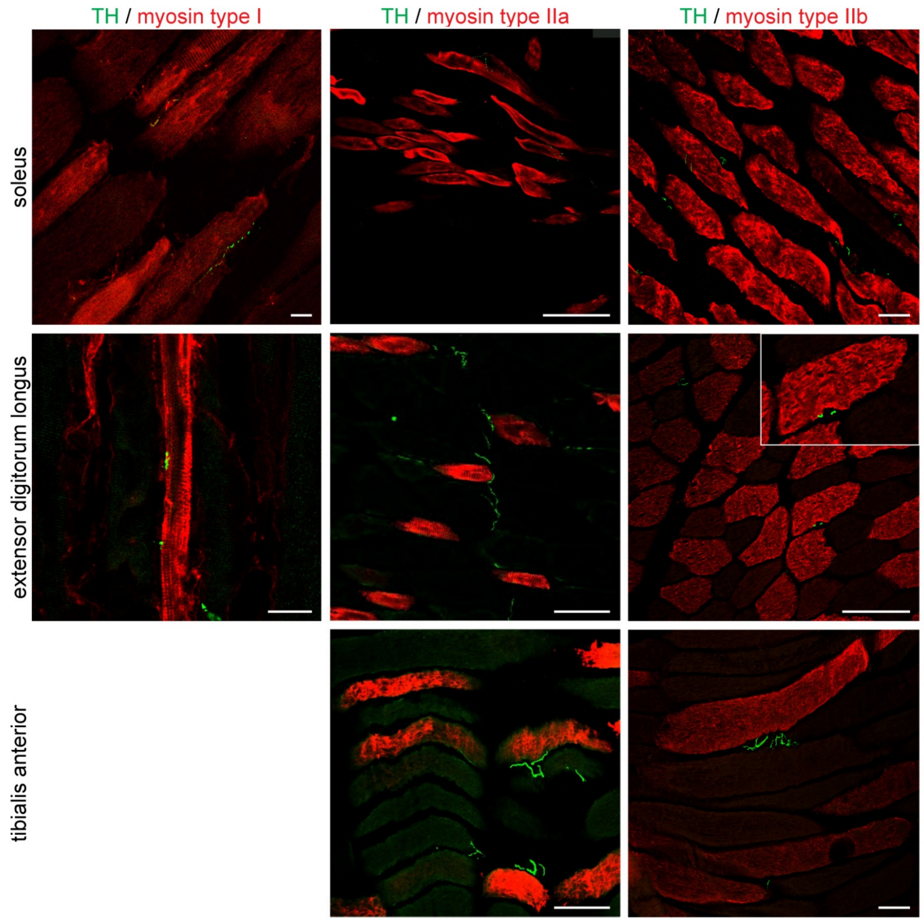

Figure S1. TH-positive axons approach slow and fast muscle fibers in different muscles. Soleus, extensor digitorum longus, and tibialis anterior muscles were taken from adult wildtype mice (P90). Muscles were washed, fixed in 1 % PFA for 15 minutes at room temperature, dehydrated in sucrose gradient and frozen in liquid nitrogen. Ten microns-thick longitudinal sections were prepared, immunostained against TH and myosin types I (left column), IIa (middle column), or IIb (right column) and then used for confocal analysis. Images show single optical sections of muscles slices and depict immunofluorescence signals of TH (green) and myosins (red). The small insert in the middle right panel is a detail of the corresponding overview image. Scalebars, 50 µm.

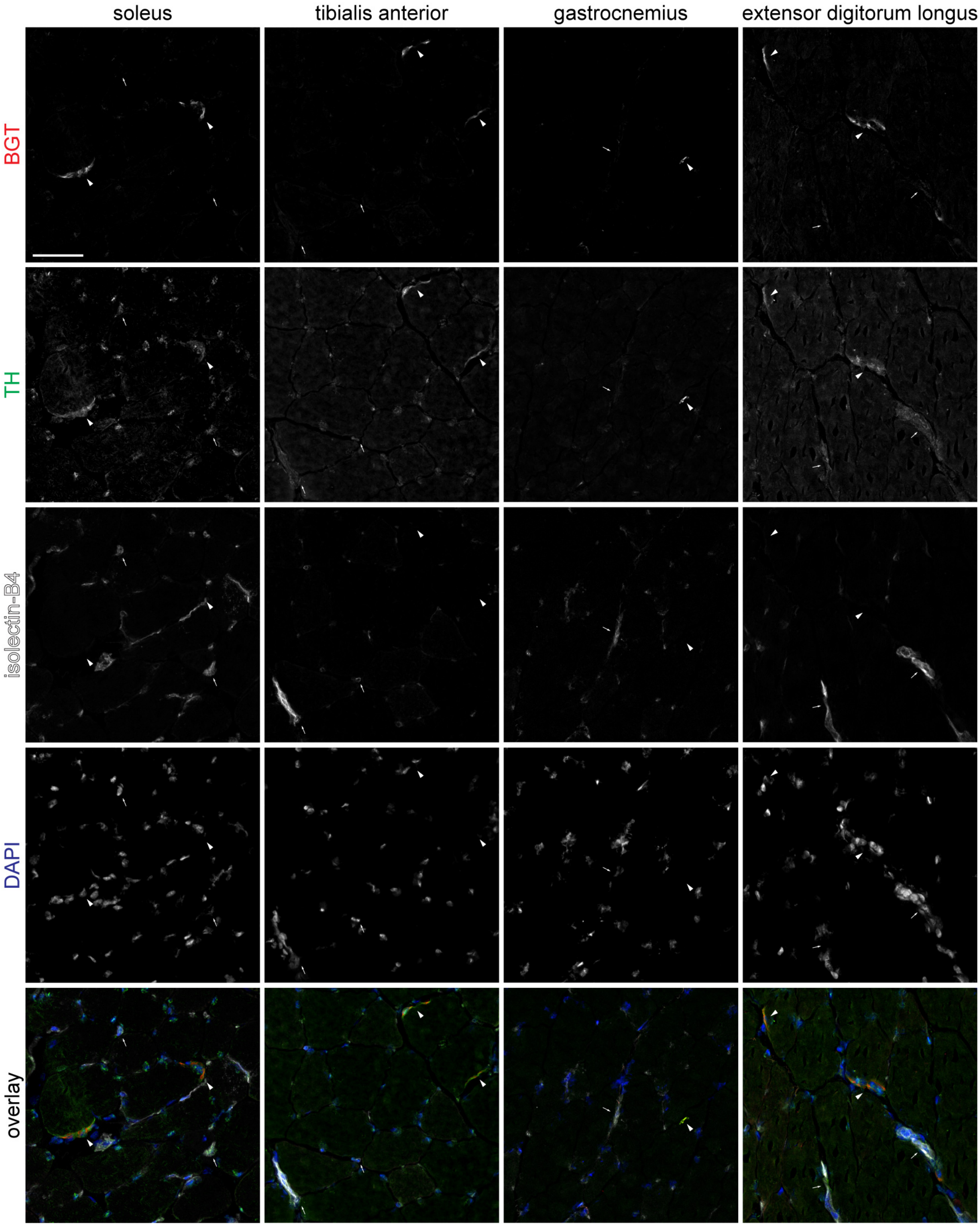

Figure S2. TH-positive structures approach NMJs and blood vessels. Soleus, tibialis anterior, gastrocnemius, and extensor digitorum longus muscles were taken from adult wildtype mice. Muscles were directly frozen after dissection. 10 µm thick transversal sections were prepared, stained against NMJs (BGT), sympathetic structures (TH), blood vessels (isolectin-B4, 1:100), and nuclei (DAPI) and then imaged by confocal microscopy. Images show maximum z-projections of representative regions. In overlay images, BGT, TH, isolectin-B4, and nuclei are depicted in red, green, white, and blue, respectively. Arrowheads and arrows point to TH-positive NMJs and blood vessels, respectively. Scalebar, 50 µm.
